# Influencing Factors of Massive Hemorrhage and High-Grade Renal Vascular Injury after PCNL: A Retrospective Comparative Study

**DOI:** 10.1155/2023/5521691

**Published:** 2023-11-24

**Authors:** Qiushi He, Ziyan Song, Xinrui Wang, Bingbing Hou, Zongyao Hao

**Affiliations:** ^1^Department of Urology, The First Affiliated Hospital of Anhui Medical University, Anhui Medical University, Hefei, China; ^2^Department of Urology, Institute of Urology, Anhui Medical University, Hefei, China; ^3^Department of Urology, Anhui Province Key Laboratory of Genitourinary Diseases, Anhui Medical University, Hefei, China; ^4^School of Life Sciences, Anhui Medical University, Hefei, Anhui, China

## Abstract

**Purpose:**

Severe hemorrhage after percutaneous nephrolithotomy (PCNL) is a rare but alerting event. In this study, we report the factors affecting massive hemorrhage after PCNL, various levels of vascular damage during renal angiography, and the therapeutic effect of superselective renal artery embolization (SRAE). *Patients and Methods*. A retrospective analysis was performed on the data of 69 patients with postoperative PCNL hemorrhage who underwent SRAE from January 2010 to March 2021. Inclusion criteria for all cases were failure of conservative treatment for severe renal hemorrhage after surgery and then treatment with SRAE. In addition, 98 patients without significant hemorrhage after PCNL were randomly selected as the control group. All clinical data are confirmed by imaging and laboratory examinations. We performed univariate and multivariate analyses to find risk factors of massive hemorrhage and high-grade renal vascular injury after PCNL.

**Results:**

A total of 69 patients underwent angiography, 64 of which received SRAE due to positive hemorrhages detected by angiography. Urinary tract infection (OR (95% CI) = 11.214 (2.804∼44.842)), high blood pressure (OR (95% CI) = 5.686 (1.401∼23.083)), and no hydronephrosis (OR (95% CI) = 0.189 (0.049∼0.724)) are the most important factors leading to massive hemorrhage after PCNL. In patients who need SRAE after hemorrhage, high-grade vascular injury (grade III) is related to advanced age and decreased hemoglobin.

**Conclusion:**

During the perioperative period of PCNL, patients with a risk of hypertension, urinary tract infection, and no hydronephrosis should be strengthened to monitor their high risk of postoperative hemorrhage. For patients with postoperative hemorrhage, we can use the patient's age and decreased hemoglobin before and after operation for analysis. In this way, individualized assessment can greatly improve the efficiency of SRAE treatment.

## 1. Introduction

Percutaneous nephrolithotomy (PCNL) is the preferred surgical procedure for the treatment of 20–40 mm renal stones with excellent stone-free rate (SFR) [1]. With the update of surgical instruments and the improvement of surgical methods, PCNL, as a safer surgical method, is increasingly used in clinical practice. However, surgical complications are still inevitable, and hemorrhage is the most common and serious one. Although post-PCNL bleeding may not be caused by iatrogenic factors, it may still be due to damage to the renal parenchyma or microvasculature during the procedure [1, 2]. The reported transfusion rate in patients was 7%, and the incidence of embolism was 0.4% [3]. When hemorrhage occurs, experienced doctors will estimate the patient's hemorrhage volume and take conservative treatment quickly, such as intravenous infusion, local fixation, and red blood cell transfusion. However, in more than 90% of the cases, SRAE has been shown to be an effective and sometimes life-saving procedure [4–7]. By selectively placing the catheter into the corresponding blood vessel, the degree and type of the patient's blood vessel damage can be accurately judged. Meanwhile, the selection of appropriate embolization materials can achieve a successful vascular embolism rate of 89%–96% [4, 8, 9]. Previous studies have reported risk factors for initial embolization failure, such as hydronephrosis <20 mm, total ultrasonographic guidance, isolated kidney, history of previous ipsilateral renal surgery, PCNL duration >90 minutes, and multiple bleeding sites [10], but they have neglected to explore risk factors of massive hemorrhage and high-grade renal vascular injury after PCNL. In this study, we collected detailed perioperative data and specifics of vascular injuries in patients who underwent SRAE for uncontrolled bleeding due to PCNL in recent years, categorized the patients into different grades of vascular injuries, and attempted to explore the risk factors for high-grade vascular injuries with the aim of reducing the harm of vascular injuries in patients with PCNL.

## 2. Patients and Methods

### 2.1. Study Population and Design

We collected a total of 69 patients with massive hemorrhage requiring SRAE after PCNL in the First Affiliated Hospital of Anhui Medical University which is a regional medical center in East China, from January 2011 to March 2021. In the past ten years, on average, there are about 800 patients undergoing percutaneous renal puncture or PCNL in our hospital every year. In addition, 98 patients with PCNL were randomly selected as normal controls from 2011 to 2021. The inclusion criteria were patients receiving PCNL without significant bleeding or with minor bleeding that resolves with conservative treatment. The exclusion criteria were patients with renal insufficiency, congenital abnormalities, and solitary kidney. Indications for patients with postoperative massive hemorrhage that require SRAE include massive or persistent renal hemorrhage, progressive decline in hemoglobin, and shock symptoms such as paleness and cyanosis. The optimal timing of interventional therapy is determined by an experienced interventional radiologist.

### 2.2. Investigated Data

The collected patients' data mainly include patient's preoperative data, surgical data, and imaging data. Preoperative data include BMI, hydronephrosis, stones' characteristics, blood coagulation status, urinalysis and urine culture, liver and kidney function, underlying medical history, and surgical history. Surgical data include operative time, surgical side, number of accesses, path, and number of punctures. Image data include bleeding location, grade and type of renal-responsible blood vessel damage under digital subtraction angiography (DSA), and the results of embolization.

Patients with a history of hypertension were controlled to a blood pressure of 160/90 mmHg with regular use of antihypertensive medication both preoperatively and postoperatively. Patients with a history of using anticoagulant drugs such as aspirin were all stopped 5 days before surgery, and patients with preoperative urinary tract infection received treatment until urine culture was negative. The prophylactic use of broad-spectrum antibiotics was performed for patients without urinary tract infection before surgery to prevent infection. For patients diagnosed with DM before surgery, preoperative fasting blood glucose control is less than 7.8 mmol/L and postprandial blood glucose is less than 10 mmol/L. Oral hypoglycemic drugs are discontinued on the surgery day, and for patients treated with insulin, the normal basal insulin dose is given the day before and on the day of surgery. Conservative treatment for postoperative patients includes strict bed rest, adequate fluid replacement, clamping of the nephrostomy tube, and hemostatic drugs such as tranexamic acid. When conservative treatment failed, the patient was transferred to the DSA room for interventional embolization. A nephrostomy tube is routinely left in place when PCNL is performed in our center, which is to routinely remove the nephrostomy on postoperative day 2 or postoperative day 3, and when a large amount of bright red blood is seen in the nephrostomy tube, the patients were considered for delayed removal of the nephrostomy and conservative treatment measures such as clamping of the nephrostomy tube, rehydration of fluids, and application of antiemetic medication are taken immediately. In the control group, in most cases, no significant bleeding was seen in the nephrostomy tube without clamping, and in a small number of cases, bleeding stopped after conservative treatment measures such as clamping the nephrostomy tube.

### 2.3. Angiography and Embolization Technique

All embolization operatives are performed by experienced interventional radiologists. During renal artery angiography, right femoral artery was punctured using Seldinger technology, 5-F cobra catheter was used for SRAE cannulation, and iodoethanol was used as a contrast agent. After clarifying the hemorrhage site, we inserted embolic materials such as gelatin sponge or spring coil through arterial cannula and observed target angiographic-contrast agent overflow or flow stop. We repeated renal angiography to ensure complete control of hemorrhage, and technical success was defined as no evidence of contrast leakage from the aneurysmal sac or internal arteriovenous fistula (IAVF). Finally, the patient groin was dressed with pressure bandages for 24 hours to prevent hematoma.

In addition, all angiography results are analyzed by interventional radiologists. The responsible vessels are classified according to the type and location of the involved arteries. Anatomically, the renal artery is divided into the renal capsular artery, the inferior adrenal artery, and the renal pelvic ureteral artery and is divided into two terminal branches, namely, the anterior branch and the posterior branch step by step, dividing into segment arteries, interlobar arteries, and interlobular arteries, respectively. According to the course and size of renal blood vessels, the renal arteries are divided into 6 grades. The grade I branch is the renal artery, the grade II is the main artery in the front or back of the renal artery branch, segmental arteries are grade III branches, interlobar arteries are grade IV branches, and grade V branches and grade VI branches are arcuate arteries and interlobular arteries, respectively. In addition, arteriovenous fistula is mainly due to the intercommunication of the arteriovenous system and the early filling of the venous system during renal arteriography. Pseudoaneurysms are considered to be cystic dilation of arterial branches. Renal artery branch tears at all levels are characterized by the overflow of the contrast agent at the end of the arterial branch. After the operation, the hemorrhage vessel damage and the type of embolization material were recorded.

### 2.4. Statistical Analysis

To assess the risk factors of postoperative hemorrhage and different grades of vascular injury in patients with PCNL, a chi-square test was used for categorical variables and the Mann–Whitney *U* test and the Kruskal–Wallis test were used for continuous variables. We retrospectively analyzed multiple factors in these groups, including patient and kidney characteristics, stone burden, PCNL technique, and SRAE procedure. Statistical analysis was done using SPSS (version 20.0), and *P* < 0.05 was considered statistically significant.

## 3. Results

### 3.1. Patient Characteristics and Angiography Findings for Hemorrhage after PCNL

About 8,000 cases of PCNL have been completed in our center in the last 10 years, a total of 69 PCNL patients underwent SRAE; the incidence of hemorrhage after PCNL and requiring SRAE is approximately 0.86%. 5 of them had no hemorrhage after angiography, and no special treatment was given. Among the 69 patients, there were 56 males (81.1%) and 13 females (18.9%), with an average age of 49.7 years (19–78 years). None of the abovementioned cases had serious complications such as nephrectomy or death after surgery. In the remaining 64 patients, 6 patients suffered from upper renal vascular injury, 44 patients suffered from middle vascular injury, 6 patients suffered from lower vascular injury, 5 patients suffered from middle and upper vascular injury, and 3 patients suffered from middle and lower vascular injury. Among the patients with vascular injury confirmed by angiography, 38 patients (59.4%) had pseudoaneurysm formation, 8 patients (12.5%) had arteriovenous fistula, 8 patients (12.5%) had pseudoaneurysm with arteriovenous fistula, and 10 patients (15.6%) had arterial rupture. The mean duration between PCNL and the development of hemorrhage in these patients were 12.37 d, 10.75 d, 7.5 d, and 14.5 d, respectively. The vascular damage of the patients caused by puncture is mainly concentrated in grade 3–5 blood vessels, 15 cases (24.3%) had grade three vascular injury, 44 cases (68.2%) had grade four vascular injury, and 5 cases (7.5%) had grade five vascular injury.

**Figure 1:**
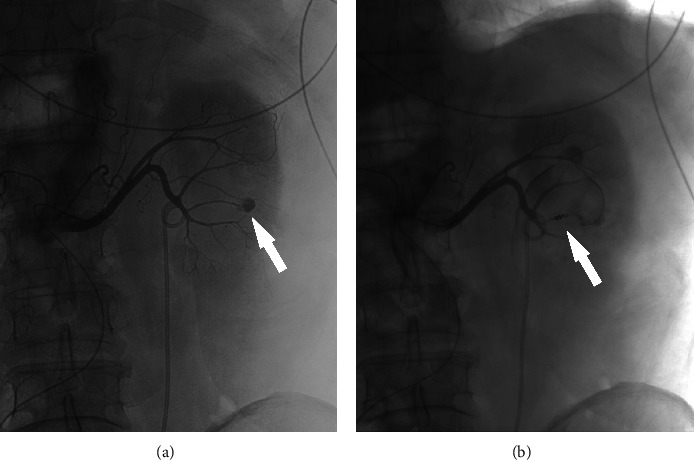
(a) Pseudoaneurysm formation after interlobular artery rupture (white arrow). (b) After the embolization was completed, the arrow shows that the coil was placed and no contrast medium exuded at the hemorrhage point.

**Figure 2:**
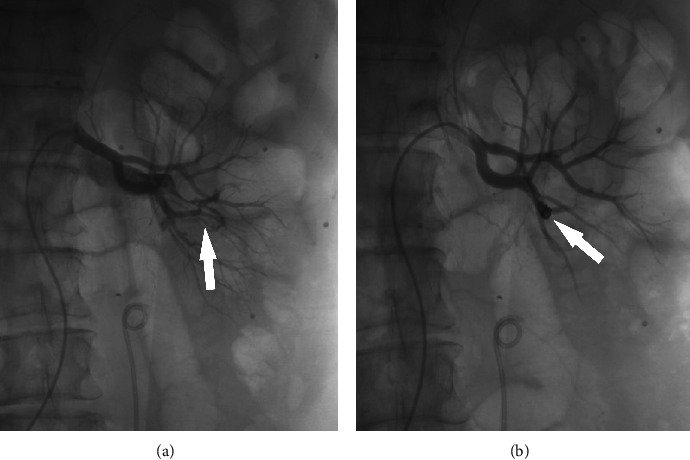
(a) The interlobar artery was ruptured and connected to segmental artery, forming an arteriovenous fistula (white arrow). (b) The arrow shows that the arteriovenous fistula was completely occluded by 2 microcoils and the mixture of tissue glue and poppy lipiodol.

**Figure 3:**
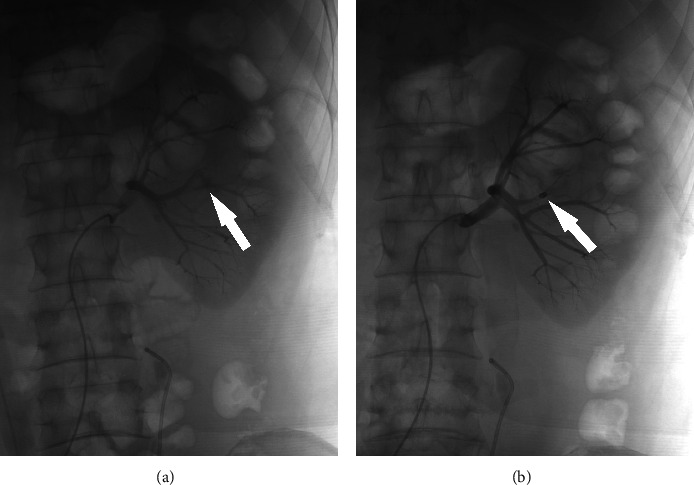
(a) An arcuate artery was ruptured (white arrow). (b) The arrow shows that the vascular break site was completely occluded by 1 sponge.

The abovementioned patients were treated with embolization in time according to different types of vascular injury. Embolization materials include gelatin sponge, iodized oil, PVA particles, and spring coils. After embolization, reimaging showed that hemorrhage stopped immediately. The following is a comparison of three representative groups before and after embolization (Figures 1–3).

### 3.2. Risk Factors for Postoperative Hemorrhage after PCNL

A total of 69 SRAE cases and 98 control cases without postoperative hemorrhage were included in this study. For their general characteristics, see Tables 1 and 2. The results of the Mann–Whitney *U* test for continuous variables and the chi-square test for categorical variables showed that the number of multiple tracts, urinary tract infection, diabetes, hypertension, no hydronephrosis, and low glomerular filtration rate in affected kidney are suspected influencing factors of intraoperative and postoperative hemorrhage in PCNL.

The regression variable was whether there was hemorrhage or not, and the independent variable was the number of accesses, urinary tract infection, diabetes, hypertension, hydronephrosis, and filtration rate of the affected kidney. *P* ≤ 0.05 was used as criteria for the selection of variables. Multivariate logistic regression analysis showed that number of accesses, urinary tract infection, hypertension, and no hydronephrosis were risk factors for intraoperative high-grade vascular injury hemorrhage after percutaneous nephrolithotomy. See Table 3 for details.

### 3.3. Influencing Factors of High-Grade Vascular Injury in Percutaneous Nephrolithotomy

Excluding 5 people whose vascular injury grade is difficult to define, a total of 64 postoperative hemorrhage cases that meet requirements are divided into 3 groups according to the vascular injury grade, and their general characteristics are shown in Tables 4 and 5. The results of the Kruskal–Wallis test for continuous variables and the chi-square test for categorical variables showed that the operation time, age, decreased hemoglobin, diabetes, and history of ipsilateral kidney surgery are suspected influencing factors that cause hemorrhage of high-grade vessels that are damaged during PCNL.

The regression variable was whether there was bleeding or not, with *P* ≤ 0.05 as the independent variable selection criterion, and operative time of surgery, age, decreased hemoglobin, diabetes mellitus, and history of ipsilateral kidney surgery were included as independent variables. After multivariate logistic regression analysis, it was shown that the main risk factors for grade 3 vascular injury compared to grade 4 vascular injury and grade 5 vascular injury were age and decreased hemoglobin, as detailed in Table 6.

## 4. Discussion

Compared with the traditional open method, PCNL has advantages of faster postoperative recovery and shorter hospital stay, but there are still complications, among which the most common complication is hemorrhage [11], which should be paid special attention by clinicians. It has been reported that hemoglobin of patients can decrease by 31.0–79.0 g/L [12], which will have a huge adverse effect on patient and postoperative recovery of renal function. If there is hemorrhage during operation, large-scale nephrectomy or suture of suspected hemorrhage is a commonly used method in the past, but it is very harmful to patient's renal function [13]. Therefore, research on the risk factors of hemorrhage during PCNL surgery can help prevent key factors in clinical diagnosis and treatment process in advance.

In our study, we found that hypertension, urinary tract infection, and absence of hydronephrosis were associated with hemorrhage complications. The incidence of hypertension in patients with severe hemorrhage after PCNL was 42.2%, which was almost double the 21.4% in the control group. In our experience, hypertension should be a predictor of severe hemorrhage after PCNL, but when we compared with previous studies, different conclusions emerged. Said et al. [14] pointed out that hypertension has no effect on hemorrhage after PCNL. However, this study found that patients with hypertension have a higher risk of hemorrhage, which may be related to the sclerosis of renal arterioles, loss of elasticity, and hyalinization caused by hypertension, which increases the risk of hemorrhage. In addition, urinary tract infection can stimulate patient's own immune system, resulting in the release of inflammatory mediators into the blood system, such as plasminogen activator, which can activate the fibrinolytic system and lead to enhanced fibrinolytic activity and hemorrhage in the perioperative period.

The reports of Lee et al. [15] and Kim et al. [16] have a similar view that a lack of hydronephrosis was an important risk factor for transfusion in PCNL. Wang et al. [17] reported that no hydronephrosis occurred in patients with severe renal hemorrhage. A possible explanation for this finding is that when there is more hydronephrosis in the affected kidney, the renal cortex is squeezed and thinned. Compression from hydronephrosis results in the reduced renal blood flow and even ischemia, and thicker renal parenchyma in patients without hydronephrosis increases the risk of needle penetration at all levels.

With the advancement of medical imaging, superselective angiography can accurately reflect the hemorrhage site of the responsible blood vessel and the level or type of blood vessel damage. We have conducted a detailed study of vascular damage after renal puncture, and location of the puncture, use of fascial dilators, cutting effect of needle, and distribution of renal blood vessels are all important factors. In this study, 52 cases (81.3%) had vascular injury close to the midrenal segment, the same puncture site as most PCNL, and percutaneous renal access near the midkidney meant better surgical field of view and higher SFR. A needle with a sharp tip and fascial dilator damages the artery, causing it to partially or completely rupture it and may lead to renal pseudoaneurysms and leakage of contrast media, and arteriovenous fistulas (25%) may form only if renal artery and venous damage occurs at the same time [2, 18]. The results of this study suggest that the segmental artery and interlobar arteries are the most common types of injured arteries requiring embolization after PCNL. The main branches of the renal artery are close to the renal hilus and have a thick muscular layer, which is more difficult to damage. At the same time, terminal blood vessels have thin and widely distributed branches, and although they are easily damaged, massive hemorrhage rarely occurs due to low blood flow. In order to explore the causes of high-grade vascular damage such as grade III, we graded the damaged blood vessels caused by puncture and performed multivariate analysis on relevant patient's data. We found that higher-grade vascular injury (grade III) was associated with age and decreased hemoglobin before and after surgery.

Older patients are more likely to develop severe vascular damage by classifying vascular damage into different grades. With the increase of age, collagen fibers and mucopolysaccharides in the vascular wall increase, the elastic fibers of the vascular wall decrease, and calcification of the tube wall leads to reduced vascular elasticity, loss of elasticity, and hardening of blood vessels. Partial or complete rupture of the artery is more likely to occur with renal puncture or fascial dilators, which may result in a tear of the renal artery or a pseudoaneurysm of the kidney [18]. Next, decreased hemoglobin usually corresponds to higher levels of vascular damage, which is an important reference indicator for timely determination of bleeding.

The results of this study can provide some guidance for doctors. If the patient has a history of hypertension, auxiliary examination shows urinary tract infection and no hydronephrosis and postoperative hemorrhage should be noted. Considering the harm and time urgency of hemorrhage to patients, it is necessary to improve the treatment efficiency of SRAE. When PCNL patients have massive hemorrhage after surgery, we can collect multiple indicators such as age and decreased hemoglobin data to determine whether there is high-grade vascular injury. This study has a certain guiding significance for the preoperative evaluation, perioperative monitoring, and hemorrhage management of PCNL surgery and aims to comprehensively and completely reduce the harm of massive hemorrhage to patients with PCNL surgery.

The present study also has some limitations. First, this study is a retrospective study, which creates a certain selection bias. Second, the time span of the cases included in this study is relatively large. As interventional techniques and embolic materials continue to evolve, complete standardization cannot be achieved. Finally, as a single-center study, the sample size in this study may have influenced our findings. More rigorous results require multicenter and large-scale studies.

## 5. Conclusion

Hypertension, urinary tract infection, and no hydronephrosis are risk factors for hemorrhage complications in patients after PCNL. Such patients should be closely monitored to be aware of their high risk of postoperative hemorrhage. SRAE is a safe and effective method for the treatment of massive hemorrhage after PCNL. It can help us judge whether there is high-grade vascular damage through several indicators such as patient's age and the decreased hemoglobin before and after the PCNL operation. The implementation of individualized SRAE treatment with the abovementioned indicators is expected to improve the efficiency of interventional treatment.

## Figures and Tables

**Table 1:** Univariate analysis of risk factors for massive hemorrhage after PCNL (categorical variables).

	Control	Test	*P*
Size of sheaths (%)			0.586
F16	12 (12.3)	6 (8.7)	
F18	50 (51.0)	45 (65.2)	
F20	29 (29.6)	13 (18.9)	
F22	2 (2.0)	3 (4.3)	
F24	5 (5.1)	2 (2.9)	
Number of accesses (%)			0.006
1	96 (98)	58 (84.0)	
2	2 (2)	8 (11.6)	
3	0 (0)	2 (2.9)	
4	0 (0)	1 (1.5)	
Urinary tract infection (%)			0.004
Yes	21 (21.4)	28 (40.6)	
No	77 (78.6)	41 (59.4)	
Diabetes (%)			0.023
Yes	4 (4.1)	11 (15.9)	
No	94 (95.9)	58 (84.0)	
Hypertension (%)			0.010
Yes	21 (21.4)	29 (42.0)	
No	77 (78.6)	40 (58.0)	
Surgical side (%)			0.098
Left	52 (53.1)	44 (63.7)	
Right	46 (46.9)	25 (36.2)	
Hydronephrosis (%)			<0.001
Yes	81 (82.7)	23 (33.3)	
No	17 (17.3)	46 (66.7)	
History of ipsilateral kidney surgery (%)			0.058
Yes	16 (77.6)	20 (29.0)	
No	82 (22.4)	49 (71.0)	
Multiple/staghorn stones (%)			0.492
Yes	31 (31.6)	19 (27.5)	
No	67 (68.4)	50 (72.5)	
The puncture path in a single puncture (%)			0.410
Upper	7 (7.4)	6 (9.4)	
Upper middle	4 (4.3)	5 (7.8)	
Middle	80 (80.8)	44 (68.8)	
Middle and lower	3 (3.2)	3 (4.6)	
Lower	4 (4.3)	6 (9.4)	

Data are presented as number (proportion).

**Table 2:** Univariate analysis of risk factors for massive hemorrhage after PCNL (continuous variables).

	Control	Test	*P* value
BMI (kg/m^2^)	98 (23.1712 ± 3.23159)	69 (23.4697 ± 3.06409)	0.326
Operative time (min)	98 (116.5306 ± 34.41086)	69 (130.9375 ± 43.30471)	0.107
Age (yr)	98 (49.7857 ± 11.54485)	69 (51.3125 ± 13.37241)	0.467
Stone size (cm)	98 (2.2816 ± 1.03718)	69 (2.5641 ± 1.09448)	0.726
Glomerular filtration rate (GFR) (ml/min)	98 (35.8458 ± 13.89348)	69 (22.5424 ± 20.48616)	0.004

Data are presented as the mean (standard deviation); BMI = body mass index.

**Table 3:** Multivariate analysis of risk factors for massive hemorrhage after PCNL.

	*β*	SE	Wald*χ*^2^	*P*	OR	95% CI
Number of accesses	−1.694	0.973	3.029	0.082	0.184	0.027∼1.238
Urinary tract infection	2.417	0.707	11.683	0.001	11.214	2.804∼44.842
Diabetes	−0.351	1.118	0.098	0.754	0.704	0.079∼6.298
Hypertension	1.738	0.715	5.911	0.015	5.686	1.401∼23.083
Hydronephrosis	−1.668	0.686	5.907	0.015	0.189	0.049∼0.724
Glomerular filtration rate (GFR)	0.024	0.019	1.498	0.221	1.024	0.986∼1.603

**Table 4:** Univariate analysis of risk factors for high-grade vascular injury (categorical variables).

	Grade III	Grade IV	Grade V	*P* value
Size of sheaths (%)				0.382
F16	1 (6.7)	4 (9.1)	1 (20.0)	
F18	12 (80.0)	28 (63.6)	1 (20.0)	
F20	2 (13.3)	9 (20.5)	2 (40.0)	
F22	(0)	2 (4.5)	0 (0)	
F24	0 (0)	1 (2.3)	1 (20.0)	
Number of accesses (%)				0.572
1.00	11 (73.3)	37 (84.1)	4 (80)	
2.00	2 (13.3)	5 (11.4)	1 (20)	
3.00	1 (6.7)	2 (4.5)	0 (0)	
4.00	1 (6.7)	0 (0)	0 (0)	
Urinary tract infection (%)				0.841
Yes	5 (33.3)	19 (43.2)	2 (40.0)	
No	10 (66.7)	25 (56.8)	3 (60.0)	
Diabetes (%)				0.020
Yes	6 (40.0)	4 (9.1)	0 (0)	
No	9 (60.0)	40 (90.9)	5 (100)	
Hypertension (%)				0.714
Yes	7 (46.7)	19 (43.2)	1 (20.0)	
No	8 (53.3)	25 (56.8)	4 (80.0)	
Surgical side (%)				0.474
Left	9 (60.0)	27 (61.4)	5 (100)	
Right	6 (40.0)	17 (38.6)	0 (0)	
Hydronephrosis (%)				0.686
Yes	4 (26.7)	16 (36.4)	1 (20.0)	
No	11 (93.3)	28 (63.6)	4 (80.0)	
History of ipsilateral kidney surgery (%)				0.049
Yes	8 (53.3)	11 (25.0)	0 (0)	
No	7 (46.7)	33 (75.0)	5 (100)	
Multiple/staghorn stones (%)				0.661
Yes	3 (20.0)	13 (29.5)	2 (40.0)	
No	12 (80.0)	31 (70.5)	3 (60.0)	
The puncture path in a single puncture (%)				0.406
Upper	2 (13.3)	3 (6.8)	1 (20.0)	
Upper middle	2 (13.3)	3 (6.8)	0 (0)	
Middle	7 (46.7)	33 (75)	4 (80)	
Middle and lower	1 (6.7)	2 (4.6)	0 (0)	
Lower	3 (20.0)	3 (6.8)	0 (0)	

Data are presented as number (proportion).

**Table 5:** Univariate analysis of risk factors for high-grade vascular injury (continuous variables).

	Grade III	Grade IV	Grade V	*P* value
BMI	15 (23.3479 ± 2.75715)	44 (23.4528 ± 3.32105)	5 (23.9844 ± 1.51947)	0.923
Operative time	15 (107.3333 ± 40.26105)	44 (137.0455 ± 41.51657)	5 (148.0000 ± 49.6991)	0.045
Age	15 (59.8667 ± 13.33560)	44 (50.2045 ± 11.90775)	5 (35.4000 ± 8.08084)	0.001
Stone size	15 (2.1800 ± 0.77478)	44 (2.6909 ± 1.20499)	5 (2.6000 ± 0.65192)	0.299
Glomerular filtration rate (GFR)	15 (26.9350 ± 24.42227)	44 (20.5133 ± 19.56481)	5 (28.9750 ± 22.26962)	0.601
Red blood cell count drop	15 (1.0722 ± 0.36745)	44 (1.2355 ± 0.48363)	5 (1.4660 ± 0.20936)	0.297
Decreased hemoglobin	15 (42.6000 ± 10.23858)	44 (27.5909 ± 10.53304)	5 (16.0000 ± 7.38241)	0.002
Decreased HCT	15 (12.4000 ± 6.21369)	44 (11.1916 ± 4.50993)	5 (11.5200 ± 6.28228)	0.808

Data are presented as the mean (standard deviation); BMI = body mass index; HCT = hematocrit.

**Table 6:** Multivariate analysis of risk factors for high-grade vascular injury.

	*β*	SE	Wald*χ*2	*P*	OR	95% CI
Grade IV						
Decreased hemoglobin	−0.207	0.073	8.110	**0.004**	0.813	0.705∼0.938
Operative time	0.023	0.015	2.524	0.112	1.024	0.995∼1.054
Age	−0.080	0.040	3.993	**0.046**	0.923	0.853∼0.998
Diabetes	−2.669	1.395	3.660	0.056	0.069	0.004∼1.067
History of ipsilateral kidney surgery	−2.093	1.136	3.396	0.065	0.123	0.013∼1.142
Grade V						
Decreased hemoglobin	−0.426	0.150	8.066	**0.005**	0.653	0.487∼0.876
Operative time	0.007	0.027	0.064	0.801	1.007	0.954∼1.063
Age	−0.243	0.092	7.026	**0.008**	0.784	0.655∼0.939
History of ipsilateral kidney surgery	−22.578	7117.067	0.000	0.997	1.565*E* − 10	a

a. Floating point overflow occurred while computing this statistic. Its value is therefore set to system missing.

## Data Availability

The data used to support the findings of this study are available from the corresponding author upon request.

## Abbreviations

PCNL: Percutaneous nephrolithotomy
SRAE: Super-selective renal artery embolization
SFR: Stone-free rate
DSA: Digital subtraction angiography
IAVF: Internal arteriovenous fistula
BMI: Body mass index
HCT: Hematocrit
CT: Computed tomography
MRI: Magnetic resonance imaging
TG: Triglyceride
MetS: Metabolic syndrome.
